# Supplementary material for: How do healthcare professionals on non-palliative care wards perceive quality of care in the dying phase? Personal and organizational predictors identified in a cross-sectional study
Source: PLoS One. 2025 Oct 31;20(10):e0334650. doi: 10.1371/journal.pone.0334650 (PMC12578199; doi:10.1371/journal.pone.0334650)
Supplement: S1 Appendix — (PDF) [file pone.0334650.s001.pdf]

Checklist for Reporting Results of Internet E-Surveys (CHERRIES)

Checklist Item	***Explanation***	***Page Number***
Describe survey design	Describe target population, sample frame. Is the sample a convenience sample? (In "open" surveys this is most likely.)<br>- Convenience sample. The wards that wanted to participate in the project were not randomly selected. However, the healthcare professionals on these wards that participated in the survey were not specifically chosen.	Data collection<br>Participants
IRB approval	Mention whether the study has been approved by an IRB.<br>- The ethics committee of the Medical Faculty of the University of Cologne (20-1727) and the General Medical Council Hamburg (2021-200061-BO-bet) approved the study, as well as all relevant staff councils at the two university medical centers.<br>- The study has been registered at the German Clinical Trials Register (DRKS: DRKS00025405).	Data collection
Informed consent	Describe the informed consent process. Where were the participants told the length of time of the survey, which data were stored and where and for how long, who the investigator was, and the purpose of the study?<br>- Informed consent information was given on the introduction page of the survey. Written consent was given via clicking the corresponding button on the introduction page to start the survey. If a potential participant did not click the continue button, we did not store any data of the participant.<br>- The introduction page stated the estimated length of the survey (10-15 minutes) and its aims. It was stated that participation is not mandatory. Ward leadership was informed about the estimated length of the survey in the project kick-off meeting.<br>- The introduction page provided hyperlinks to LimeSurvey-specific data privacy and information regarding the European General Data Protection Regulation (GDPR) for further information.	Data collection
Data protection	If any personal information was collected or stored, describe what mechanisms were used to protect unauthorized access.<br>- Data was collected via LimeSurvey and exported to SPSS- and Microsoft Excel-files and stored/archived accordingly on clinic intern servers. A consultation with the data protection officer of one of the medical centers took place before the survey was constructed to assure valid data privacy protection.	Data collection
Development and testing	State how the survey was developed, including whether the usability and technical functionality of the electronic questionnaire had been tested before fielding the questionnaire.	Description of the survey<br>Data collection

	- The questionnaire was tested by members of the research team before the data collection. On the one hand by testing the technical aspects of the questionnaire on different operating systems and internet browsers, and on the other hand by testing the methodical quality of the items of the questionnaire via pre-test with a convenience sample of n=6 healthcare professionals on the palliative care unit of the University Medical Center Hamburg-Eppendorf.	
Open survey versus closed survey	An "open survey" is a survey open for each visitor of a site, while a closed survey is only open to a sample which the investigator knows (password-protected survey).<br>- The questionnaire was technically an open survey but was sent exclusively as a link by mail to the respective ward leadership, which is why it was effectively a closed survey without a password. The survey was not published publicly. It cannot be assumed that unauthorized persons filled out the questionnaire. If unauthorized participants filled out the survey, this would have been detected within the validity checks. Participation in the survey was voluntary and no incentives were issued for participation.	Data collection
Contact mode	Indicate whether or not the initial contact with the potential participants was made on the Internet. (Investigators may also send out questionnaires by mail and allow for Web-based data entry.)<br>- The participants were not contacted directly by the research team, but by their ward leadership via e-mail. However, participants had the possibility to contact the research team if they had questions about the survey.	
Advertising the survey	How/where was the survey announced or advertised? Some examples are offline media (newspapers), or online (mailing lists – If yes, which ones?) or banner ads (Where were these banner ads posted and what did they look like?). It is important to know the wording of the announcement as it will heavily influence who chooses to participate. Ideally the survey announcement should be published as an appendix.<br>- The staff survey was announced at an initial kick-off meeting with the respective ward leadership regarding the project. The survey was sent by e-mail (survey link) to the 10 ward leaders (physicians and nurses) who sent the survey within 2-3 days to the respective employees via internal mail distribution lists. Additionally, posters informing the healthcare professionals about the survey and relating it to the project, with QR codes for the survey link, were hung on the participating wards.	Data collection
Web/E-mail	State the type of e-survey (eg, one posted on a Web site, or one sent out through e-mail). If it is an e-mail survey, were the responses entered manually into a database, or was there an automatic method for capturing responses?	Data collection

	- The LimeSurvey questionnaire was sent via E-Mail as a hyperlink by the ward leadership to potential participants. Additionally, the survey could be accessed via QR codes on posters on the respective wards. The survey link was not posted on a public website.	
Context	Describe the Web site (for mailing list/newsgroup) in which the survey was posted. What is the Web site about, who is visiting it, what are visitors normally looking for? Discuss to what degree the content of the Web site could pre-select the sample or influence the results. For example, a survey about vaccination on a anti-immunization Web site will have different results from a Web survey conducted on a government Web site<br>- Does not apply since the survey was not posted on a web site.<br>- The mailing lists were internal mailing (team members) lists from the participating wards.	
Mandatory/voluntary	Was it a mandatory survey to be filled in by every visitor who wanted to enter the Web site, or was it a voluntary survey?<br>- It was a voluntary survey. However, it was only sent to members of the respective wards, and the posters were put up on their wards. Unauthorized persons only had very limited access to the survey. It is very unlikely, that unauthorized persons participated in the survey.	
Incentives	Were any incentives offered (eg, monetary, prizes, or non-monetary incentives such as an offer to provide the survey results)?<br>- A presentation of the ward-specific survey results was offered systematically for each respective ward. Actual incentives were not offered, however.	
Time/Date	In what timeframe were the data collected?<br>- The survey was constructed to take approximately 10-15 minutes to be completed. Pre-testing showed that completion is possible in 5 minutes. The median completion time of the actual data collection was 9 minutes and 36 seconds.<br>- Data collection took place from September 2021 – December 2021. The earliest completed survey is from 10th of September – the latest from 8th of December.	Data collection
Randomization of items or questionnaires	To prevent biases items can be randomized or alternated.<br>- There was no randomization of the items or item batteries. We did not anticipate a high risk of bias in not randomizing the items.	
Adaptive questioning	Use adaptive questioning (certain items, or only conditionally displayed based on responses to other items) to reduce number and complexity of the questions.<br>- Attention was paid to survey economy (e.g. instead of using many and long validated instruments to assess the quality of care in the dying phase, we used a self-constructed item. In our opinion, this	

	did not only have positive effects for survey economy, but also reduced the risk of bias (social desirability)).<br>- No filters were used, as they weren't necessary and the complexity of the survey was not high.	
Number of Items	What was the number of questionnaire items per page? The number of items is an important factor for the completion rate.<br>- The number of items per page varied between 2 to 19	
Number of screens (pages)	Over how many pages was the questionnaire distributed? The number of items is an important factor for the completion rate.<br>- 8 pages (excluding the introduction page and the farewell page)	
Completeness check	It is technically possible to do consistency or completeness checks before the questionnaire is submitted. Was this done, and if "yes", how (usually JAVAScript)? An alternative is to check for completeness after the questionnaire has been submitted (and highlight mandatory items). If this has been done, it should be reported. All items should provide a non-response option such as "not applicable" or "rather not say", and selection of one response option should be enforced<br>- Completeness check was operationalized by making every item mandatory. Incomplete surveys were excluded from final data analysis.	Data collection
Review step	State whether respondents were able to review and change their answers (eg, through a Back button or a Review step which displays a summary of the responses and asks the respondents if they are correct).<br>- Participants could change their previous answers by navigating between the survey pages with "back"- and "forward"-buttons.	
Unique site visitor	If you provide view rates or participation rates, you need to define how you determined a unique visitor. There are different techniques available, based on IP addresses or cookies or both.<br>- Does not apply. We could only detect participants that gave written consent and therefore started the survey after the introduction page. Due to data privacy reasons, we did not collect IP addresses. Cookies prevented the repeated participation on the same web browser. We collected cookies to avoid repeated (invalid) survey participation.	Data collection
View rate (Ratio of unique survey visitors/unique site visitors)	Requires counting unique visitors to the first page of the survey, divided by the number of unique site visitors (not page views!). It is not unusual to have view rates of less than 0.1 % if the survey is voluntary<br>- Does not apply (see "Unique site visitor")	

Participation rate (Ratio of unique visitors who agreed to participate/unique first survey page visitors)	Count the unique number of people who filled in the first survey page (or agreed to participate, for example by checking a checkbox), divided by visitors who visit the first page of the survey (or the informed consents page, if present). This can also be called "recruitment" rate.<br>- Response rate: n=718 healthcare professionals were invited to the survey in total. Out of these n=718, n=254 professionals started the survey. The response rate is therefore 35%.	Data collection
Completion rate (Ratio of users who finished the survey/users who agreed to participate)	The number of people submitting the last questionnaire page, divided by the number of people who agreed to participate (or submitted the first survey page). This is only relevant if there is a separate "informed consent" page or if the survey goes over several pages. This is a measure for attrition. Note that "completion" can involve leaving questionnaire items blank. This is not a measure for how completely questionnaires were filled in. (If you need a measure for this, use the word "completeness rate".)<br>- Completion rate out of all contacted healthcare professionals is 28%. Per participating ward, this number differs from 16% to 67%.<br>- Completion rate out of all participants that started the survey (n=254) is 79% (n=201).	
Cookies used	Indicate whether cookies were used to assign a unique user identifier to each client computer. If so, mention the page on which the cookie was set and read, and how long the cookie was valid. Were duplicate entries avoided by preventing users access to the survey twice; or were duplicate database entries having the same user ID eliminated before analysis? In the latter case, which entries were kept for analysis (eg, the first entry or the most recent)?<br>- Cookies were collected to decrease the probability of multiple completion of the survey and to give healthcare professionals the opportunity to fulfill the survey another time. There were no detected cases of multiple completion. Using cookies seemed necessary however, since healthcare professionals might had work related emergencies or other duties and needed to complete the survey another time. We did not receive any feedback regarding problems when continuing the survey another time.	
IP check	Indicate whether the IP address of the client computer was used to identify potential duplicate entries from the same user. If so, mention the period of time for which no two entries from the same IP address were allowed (eg, 24 hours). Were duplicate entries avoided by preventing users with the same IP address access to the survey twice; or were duplicate database entries having the same IP address within a given period of time eliminated before analysis? If the latter, which entries were kept for analysis (eg, the first entry or the most recent)?<br>- IP addresses were not collected due to data privacy. We did not see any benefits that outweighed the concerns of saving IP addresses.	

Log file analysis	Indicate whether other techniques to analyze the log file for identification of multiple entries were used. If so, please describe.<br>- No log file was used. However, data validity was checked manually by researchers (NO, SK). There were no hints for multiple completion of individual participants. Cookies were collected to prevent repeated completion on the same web browser.	
Registration	In "closed" (non-open) surveys, users need to login first and it is easier to prevent duplicate entries from the same user. Describe how this was done. For example, was the survey never displayed a second time once the user had filled it in, or was the username stored together with the survey results and later eliminated? If the latter, which entries were kept for analysis (eg, the first entry or the most recent)?<br>- No log in was required. However, surveys were basically closed surveys due to the intern distribution by mailing lists. The survey was not posted on (public) websites.	
Handling of incomplete questionnaires	Were only completed questionnaires analyzed? Were questionnaires which terminated early (where, for example, users did not go through all questionnaire pages) also analyzed?<br>- Only completed surveys were analyzed. However, missing data analysis took place to describe missing data (e.g. patterns).	Data collection
Questionnaires submitted with an atypical timestamp	Some investigators may measure the time people needed to fill in a questionnaire and exclude questionnaires that were submitted too soon. Specify the timeframe that was used as a cut-off point, and describe how this point was determined<br>- Median completion time was 9:36. Completed questionnaires under 3 minutes were planned to be excluded. The fastest completion was 4:20. Therefore, no questionnaires were excluded due to fast completion time.	Data collection
Statistical correction	Indicate whether any methods such as weighting of items or propensity scores have been used to adjust for the non-representative sample; if so, please describe the methods<br>- Does not apply, since we exclusively analyzed fully completed surveys. Weighing items and propensity scores were not used or calculated.	Data collection

This checklist has been modified from Eysenbach G. Improving the quality of Web surveys: the Checklist for Reporting Results of Internet E-Surveys (CHERRIES). J Med Internet Res. 2004 Sep 29;6(3):e34 [erratum in J Med Internet Res. 2012; 14(1): e8.]. Article available at https://www.jmir.org/2004/3/e34/; erratum available https://www.jmir.org/2012/1/e8/. Copyright ©Gunther Eysenbach. Originally published in the Journal of Medical Internet Research, 29.9.2004 and 04.01.2012.

This is an open-access article distributed under the terms of the Creative Commons Attribution License (https://creativecommons.org/licenses/by/2.0/), which permits unrestricted use, distribution, and reproduction in any medium, provided the original work, first published in the Journal of Medical Internet Research, is properly cited.
