# Supplementary material for: How do healthcare professionals on non-palliative care wards perceive quality of care in the dying phase? Personal and organizational predictors identified in a cross-sectional study
Source: PLoS One. 2025 Oct 31;20(10):e0334650. doi: 10.1371/journal.pone.0334650 (PMC12578199; doi:10.1371/journal.pone.0334650)
Supplement: S2 Appendix — (PDF) [file pone.0334650.s002.pdf]

STROBE Statement—Checklist of items that should be included in reports of ***cross-sectional studies***

	Item No	Recommendation	Page No
**Title and abstract**	1	(*a*) Indicate the study's design with a commonly used term in the title or the abstract<br>How do healthcare professionals on non-palliative care wards perceive quality of care in the dying phase? Personal and organizational predictors identified in a cross-sectional study	Title
		(*b*) Provide in the abstract an informative and balanced summary of what was done and what was found<br>Abstract is included	Abstract
**Introduction**			
Background/rationale	2	Explain the scientific background and rationale for the investigation being reported<br>Further information in the Introduction chapter.	Introduction
Objectives	3	State specific objectives, including any prespecified hypotheses<br>Further explanation in the Introduction chapter.	Introduction
**Methods**			
Study design	4	Present key elements of study design early in the paper<br>Further information in the Methods chapter and the Appendix 1 (CHERRIES-Guideline).	Study design
Setting	5	Describe the setting, locations, and relevant dates, including periods of recruitment, exposure, follow-up, and data collection<br>Setting and location: Healthcare professionals on ten non-palliative care hospital wards at two university medical centers in Germany. Relevant dates: First completed survey: 6th of September 2021; last completed survey 10th of December 2021. Median time to complete the full survey: 9:36 minutes. Fastest completion time was 4:20 minutes.<br><br>Further information in the Methods chapter.	Data collection
Participants	6	(*a*) Give the eligibility criteria, and the sources and methods of selection of participants<br>Further information in the Methods chapter.	Data collection
Variables	7	Clearly define all outcomes, exposures, predictors, potential confounders, and effect modifiers. Give diagnostic criteria, if applicable<br>Further information in the Methods and Results chapters.	Data analysis, Participants, Descriptive results

Data sources/ measurement	8*	For each variable of interest, give sources of data and details of methods of assessment (measurement). Describe comparability of assessment methods if there is more than one group<br>This survey is part of a single group only project.<br><br>Further information in the Methods and Results chapters.	Study design, Participants, Descriptive results
Bias	9	Describe any efforts to address potential sources of bias<br>Potential bias: healthcare professionals might have felt like the questions were quality control; healthcare professionals might have rushed through the survey and did not take enough time to reflect on the questions.<br><br>We used a very simple, self-constructed item for the assessment of "perceived quality of care" that puts the emphasis on perception and not specific quality control. We excluded participants that finished the survey in under three minutes.<br><br>Further explanations can be found in Appendix S1 (CHERRIES-Guideline), Methods/Data collection and Results/Descriptive results chapters.	Study design, Descriptive results
Study size	10	Explain how the study size was arrived at<br>Post-hoc power analysis with the obtained sample size (ICUs n=126; general wards n=75) and a power of ≥80% and a two-sided alpha of 5% showed that we could detect standardized effects (Cohen's d) of ≥0.41 (unpaired t-test statistics).	
Quantitative variables	11	Explain how quantitative variables were handled in the analyses. If applicable, describe which groupings were chosen and why<br>Age and profession variables were dichotomized to fulfil predictor-requirements in a regression analysis	Participants
Statistical methods	12	(*a*) Describe all statistical methods, including those used to control for confounding<br>Frequencies, descriptive statistics (M and SD, Range, 95%-CI) and multivariable statistics (hierarchical, linear regression model). The regression model controls for the statistical association between all predictor variables (Table 5).<br>We used several methods to check statistical requirements in our data (e.g. VIF, AIC, homoscedasticity, normal distribution of the residuals, interaction effects between profession and type of ward)<br><br>Further information in the S3 Appendix (Analysis Appendix) and in the Data analysis chapter.	Data analysis
		(*b*) Describe any methods used to examine subgroups and interactions<br>Subgroup analysis was performed in the Descriptive results chapter. Due to the heterogeneity of participating wards, we presented intensive care units and general wards as subgroups (Table 2).	Participants

		(*c*) Explain how missing data were addressed<br>Every question in the online survey was mandatory. Due to the small amount of incomplete survey data, we analysed fully completed surveys only. 79% of the participating professionals (n=254) completed the survey without missing data (n=201).<br><br>Further information in the Appendix S1 (CHERRIES-Guideline).	Data collection
		(*d*) If applicable, describe analytical methods taking account of sampling strategy<br>not applicable	
		(*e*) Describe any sensitivity analyses<br>not applicable	
**Results**			
Participants	13*	(a) Report numbers of individuals at each stage of study—eg numbers potentially eligible, examined for eligibility, confirmed eligible, included in the study, completing follow-up, and analysed<br>n=718 healthcare professionals on 10 non-palliative care hospital wards were contacted by the ward leaderships. Out of the n=718 professionals, n= 254 responded to the survey (35%). Out of the n=718 professionals contacted, n=201 (28%) completed the survey without missing data. 79% of the participating professionals (n=254) completed the survey without missing data.<br><br>Further information is reported in the Methods/Data collection chapter.	Data collection
		(b) Give reasons for non-participation at each stage:<br>Reasons for non-participation given by selected ward leadership were:<br>- high rates of sick leave<br>- workload	
		(c) Consider use of a flow diagram<br>not applicable since there is no control group (single arm).	
Descriptive data	14*	(a) Give characteristics of study participants (eg demographic, clinical, social) and information on exposures and potential confounders<br><br>Further information in the Results chapter.	Participants
		(b) Indicate number of participants with missing data for each variable of interest<br>not applicable, since we only included fully completed surveys in the analysis.	

Outcome data	15*	Report numbers of outcome events or summary measures<br>Data is shown in Figure 1 with explanatory text.	Descriptive results
Main results	16	*(a)* Give unadjusted estimates and, if applicable, confounder-adjusted estimates and their precision (eg, 95% confidence interval). Make clear which confounders were adjusted for and why they were included<br>95%-CIs for the main variables are included in Table 5.<br>Further information are included in the Results chapter.	Descriptive results, Hierarchical multiple regression,
		(*b*) Report category boundaries when continuous variables were categorized<br>Age and profession were categorized (≤50 years old and >50 years old; Nurse and Rest).	Participants
		(*c*) If relevant, consider translating estimates of relative risk into absolute risk for a meaningful time period<br>not relevant	
Other analyses	17	Report other analyses done—eg analyses of subgroups and interactions, and sensitivity analyses<br>QQ-Plot and VIF-values were checked for homoscedasticity and multicollinearity and considered acceptable. AIC was calculated for model comparison. Possible interaction effects were analysed (Profession/Type of ward).<br><br>Further Information are included in Methods/Data Analysis and Results/Hierarchical multiple regression.	Data analysis, Hierarchical multiple regression
**Discussion**			
Key results	18	Summarise key results with reference to study objectives<br>Information is included in the Results/Hierarchical multiple regression chapter (Table 5 and Table 6).	Hierarchical multiple regression
Limitations	19	Discuss limitations of the study, taking into account sources of potential bias or imprecision. Discuss both direction and magnitude of any potential bias<br>Further information in the Discussion/Strengths and Limitations chapter.	Strengths and limitations
Interpretation	20	Give a cautious overall interpretation of results considering objectives, limitations, multiplicity of analyses, results from similar studies, and other relevant evidence<br>Included in the Discussion chapter.	Discussion
Generalisability	21	Discuss the generalisability (external validity) of the study results<br>The study should be revised with a larger, independent and more balanced sample outside of pandemic context.<br><br>Included in Discussion/Strengths and limitations and Future research chapters.	Strengths and limitations, Future research
**Other information**			
Funding	22	The project was funded by the German Innovation fund, Federal Joint Committee (G-BA, 01VSF19033). The funding body plays no role in the design of the study and collection, analysis, and interpretation of data and in writing the manuscript.	

*Give information separately for exposed and unexposed groups.

Note: An Explanation and Elaboration article discusses each checklist item and gives methodological background and published examples of transparent reporting. The STROBE checklist is best used in conjunction with this article (freely available on the Web sites of PLoS Medicine at http://www.plosmedicine.org/, Annals of Internal Medicine at http://www.annals.org/, and Epidemiology at http://www.epidem.com/). Information on the STROBE Initiative is available at www.strobe-statement.org.
