# Supplementary material for: How do healthcare professionals on non-palliative care wards perceive quality of care in the dying phase? Personal and organizational predictors identified in a cross-sectional study
Source: PLoS One. 2025 Oct 31;20(10):e0334650. doi: 10.1371/journal.pone.0334650 (PMC12578199; doi:10.1371/journal.pone.0334650)

Manuscript ID: PONE-D-24-31789
Manuscript's title: How do healthcare professionals on non-palliative care wards perceive quality of care in the dying phase? Personal and organizational predictors identified in a cross-sectional study
Corresponding Author: Nikolas Oubaid

Analysis Appendix

This Appendix provides additional statistical information regarding the predictor variables, the outcome variable and statistic checks for the regression model. In detail it includes:

1. *Visual presentation of the predictor variable scales (frequency distribution, box-plots, Q-Q-plot)*
    a. *General Self-Efficacy (predictor variable)*
    b. *Thanatophobia (predictor variable)*
    c. *Interprofessional patient-centered teamwork (predictor variable)*
    d. *Burden related to care in the dying phase (predictor variable)*
    e. *Perceived quality of care in the dying phase (outcome variable)*
2. *Multiple regression model, Step 1: Analysis without organizational predictors*
3. *Correlation matrix of model variables (personal predictors, organizational predictors, outcome variable (Pearson's correlation (r))*
4. *Test for interaction effects*
    a. *Test for interaction between profession (nurse vs. rest) and type of ward (ICUs vs general wards) in regards to the outcome (general linear model)*
    b. *Mean comparison for type of ward (ICUs vs general wards) and profession (nurse vs rest) regarding the outcome*
5. *Statistic checks (normal distribution of residuals, scatter plot to check homoscedasticity) for the hierarchical multiple regression model (n=193)*

1. Visual presentation of the predictor variable scales (frequency distribution, box-plots, Q-Q-plot)
a. General Self-Efficacy (predictor variable):

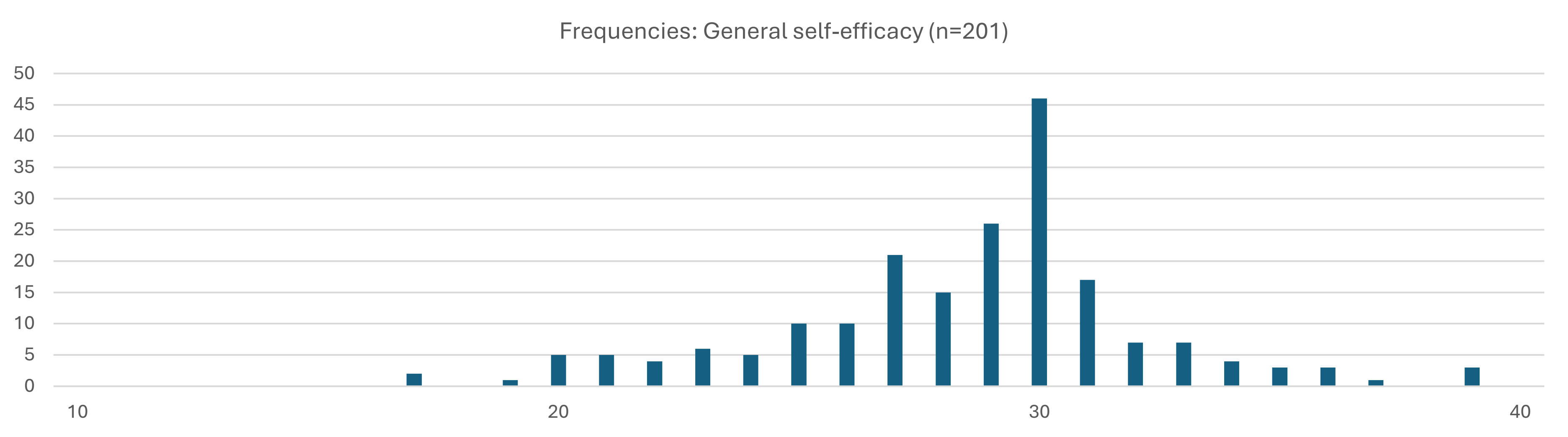

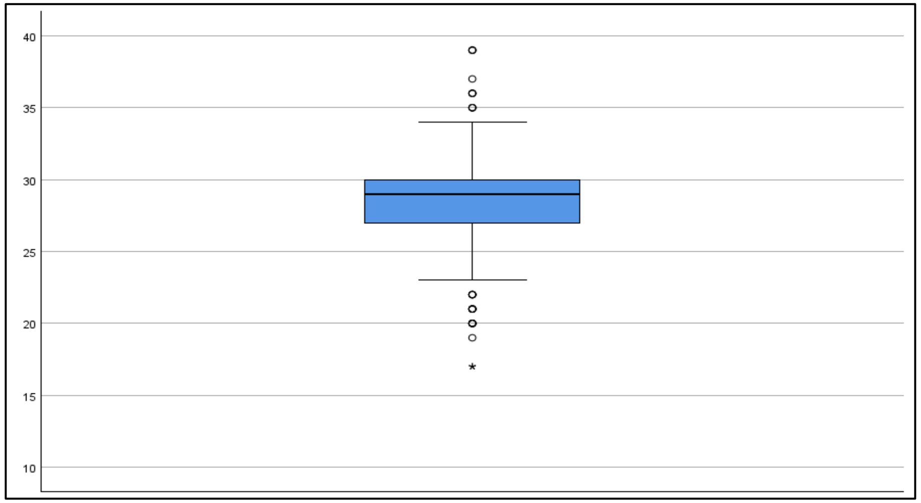

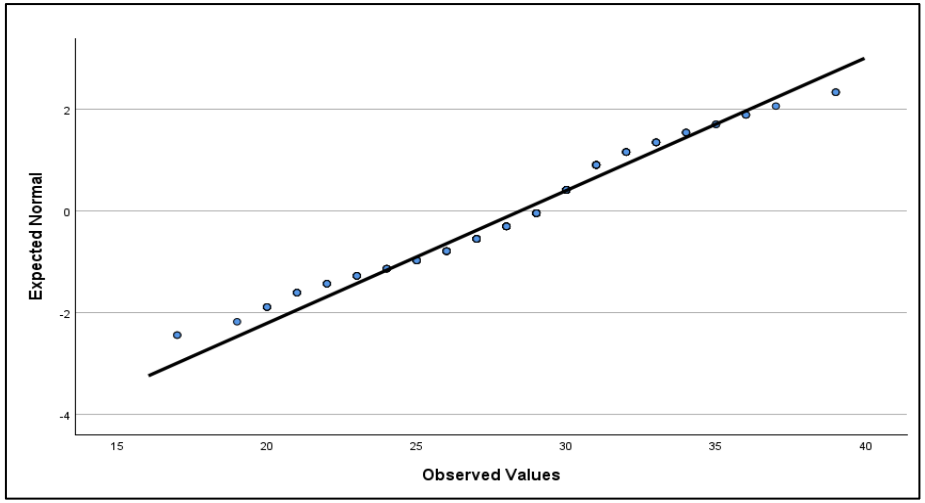

b. Thanatophobia (predictor variable):

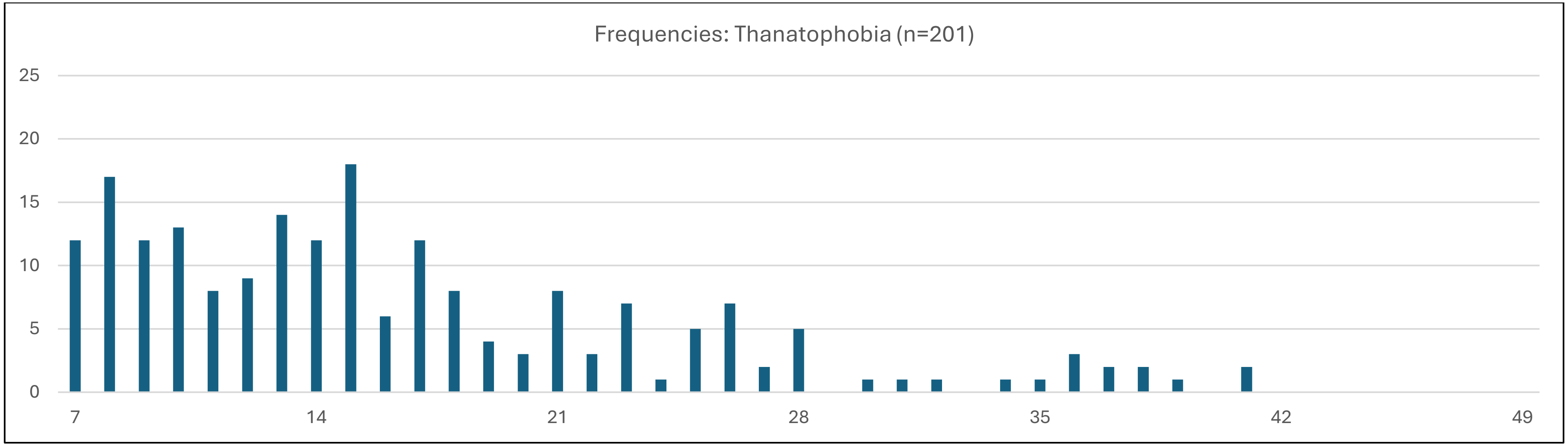

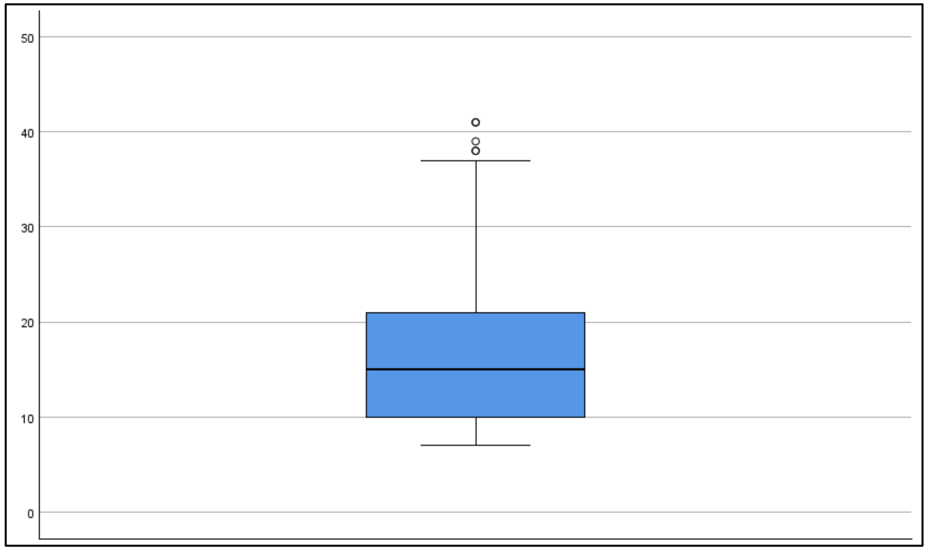

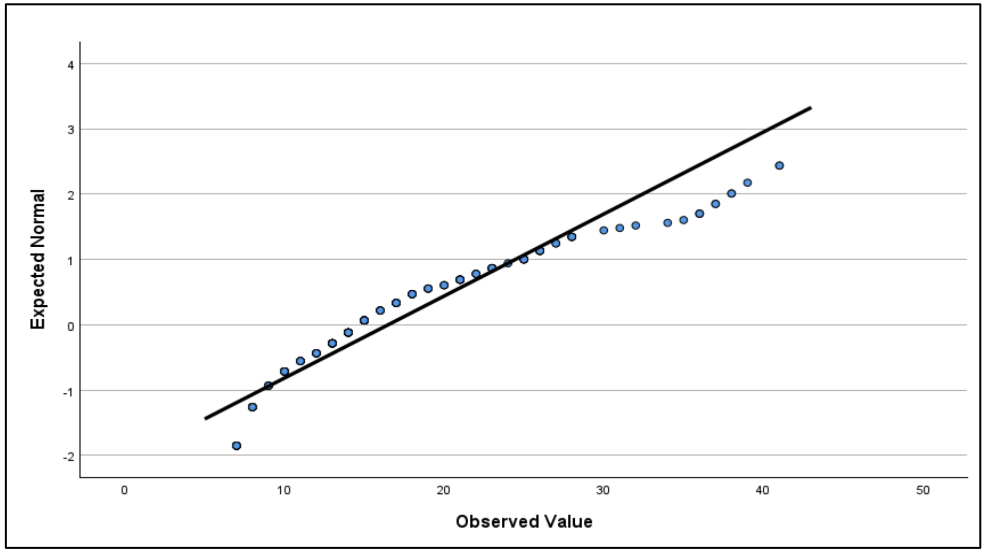

c. Interprofessional patient-centered teamwork (predictor variable):

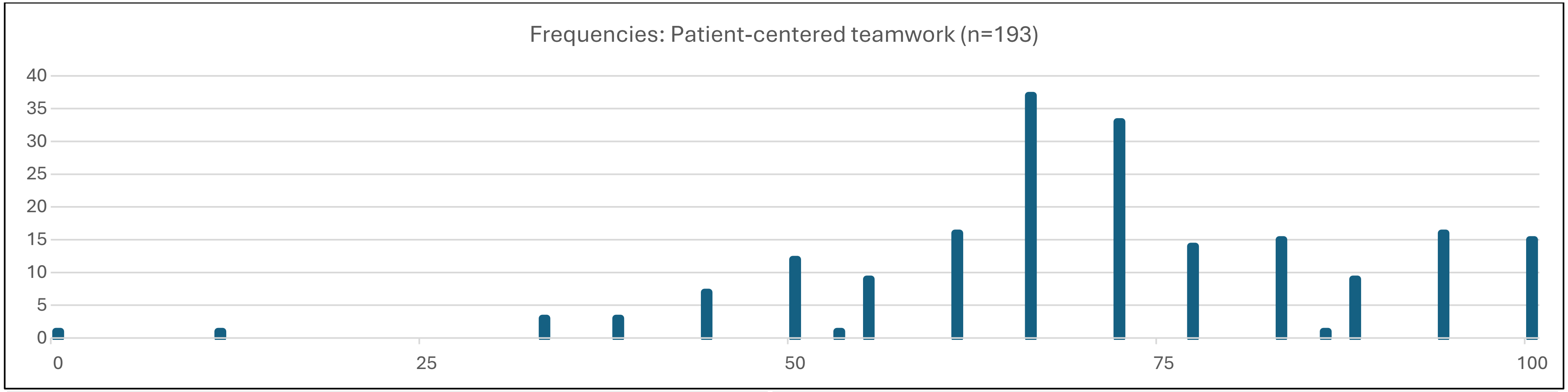

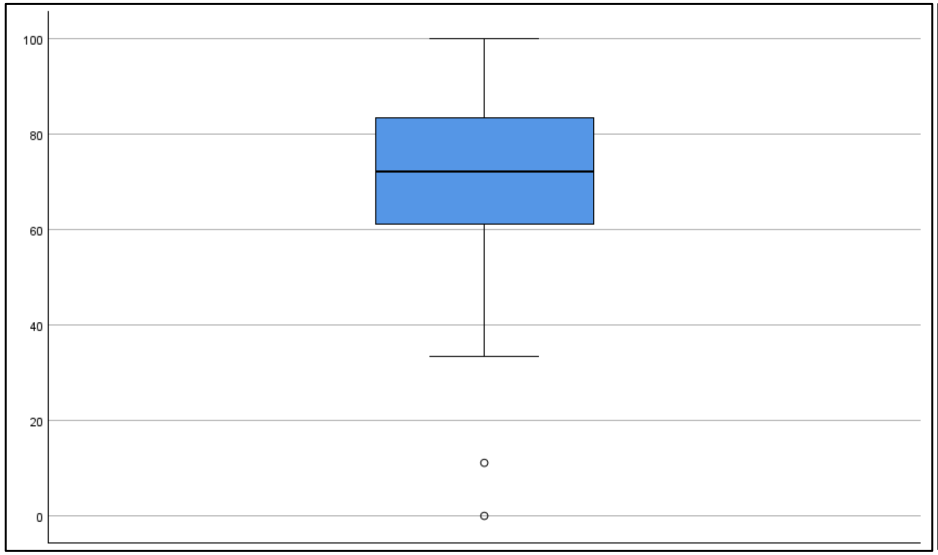

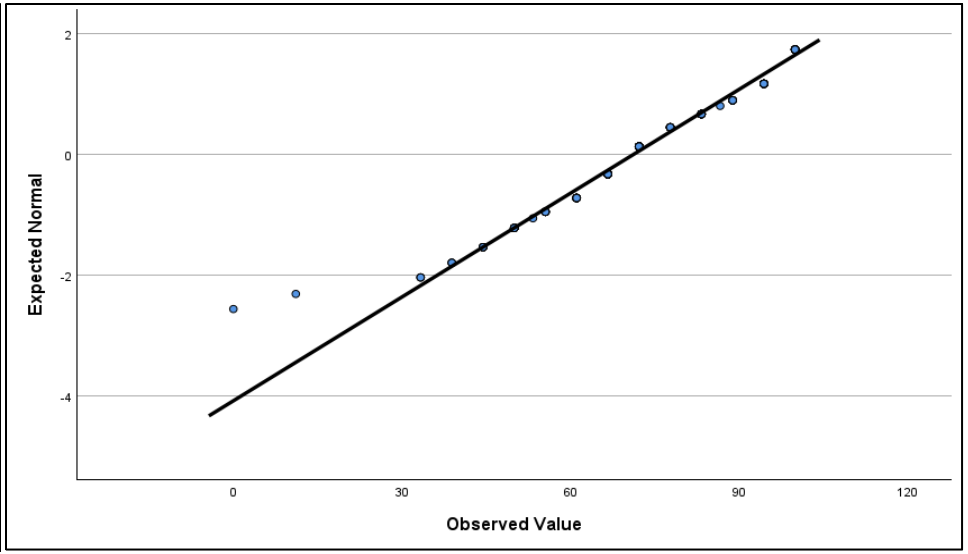

d. Burden related to care in the dying phase (predictor variable):

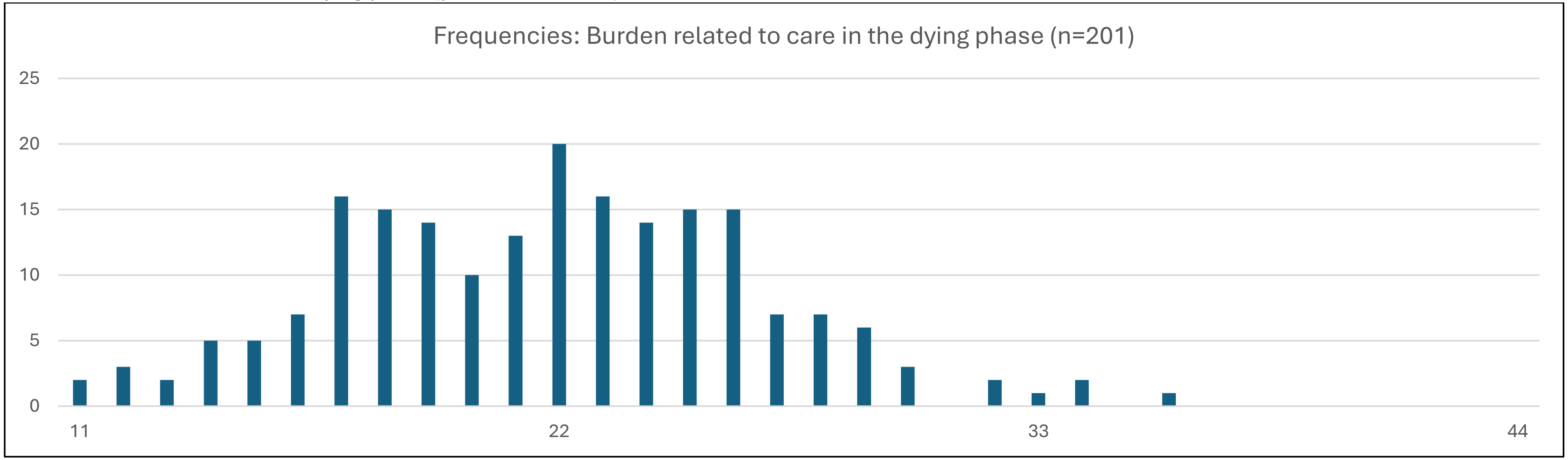

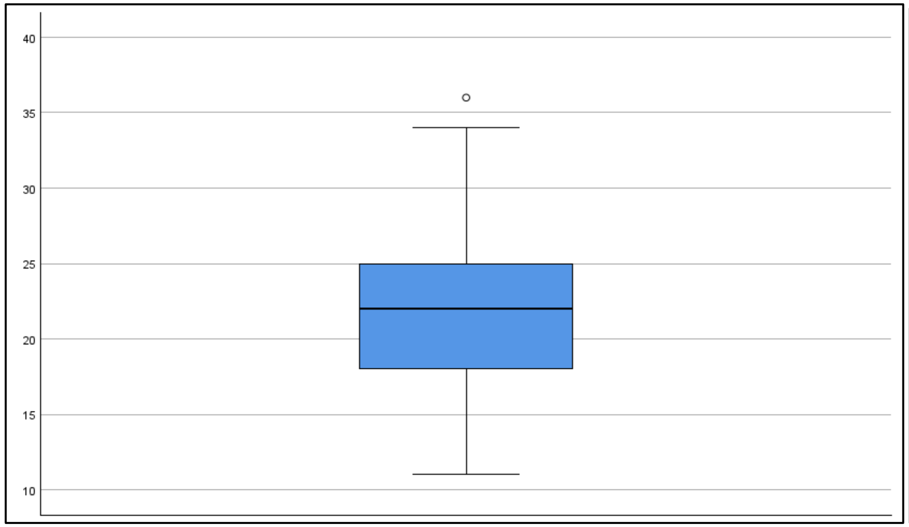

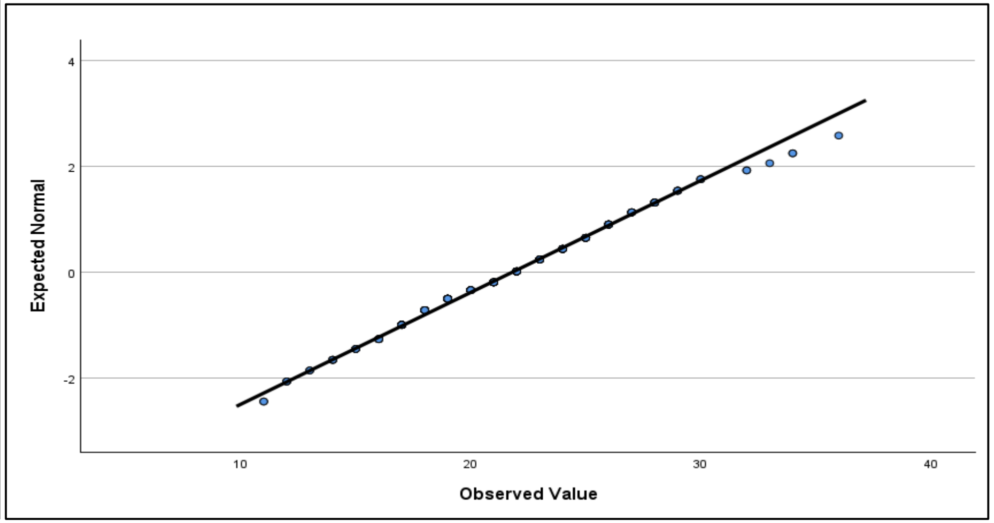

e. Perceived quality of care in the dying phase (outcome variable):

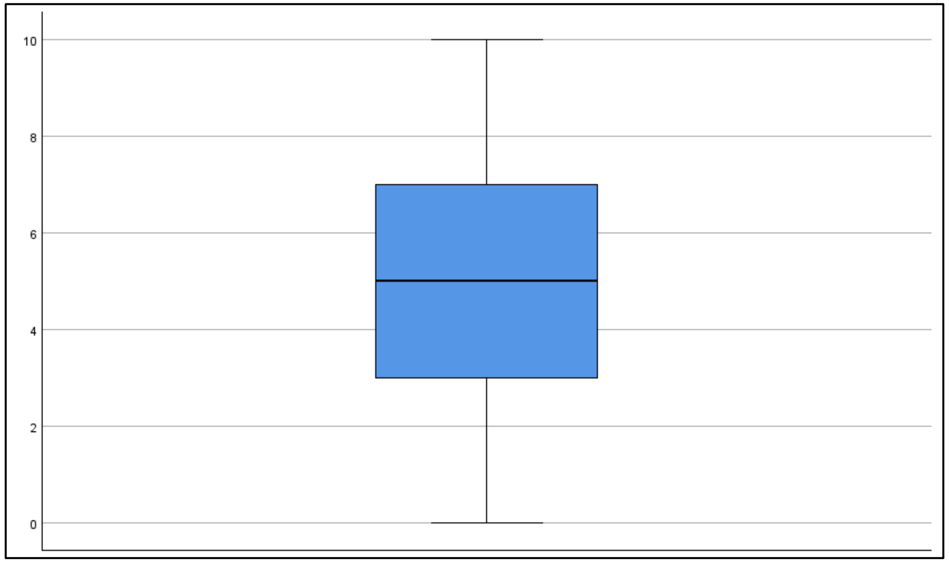

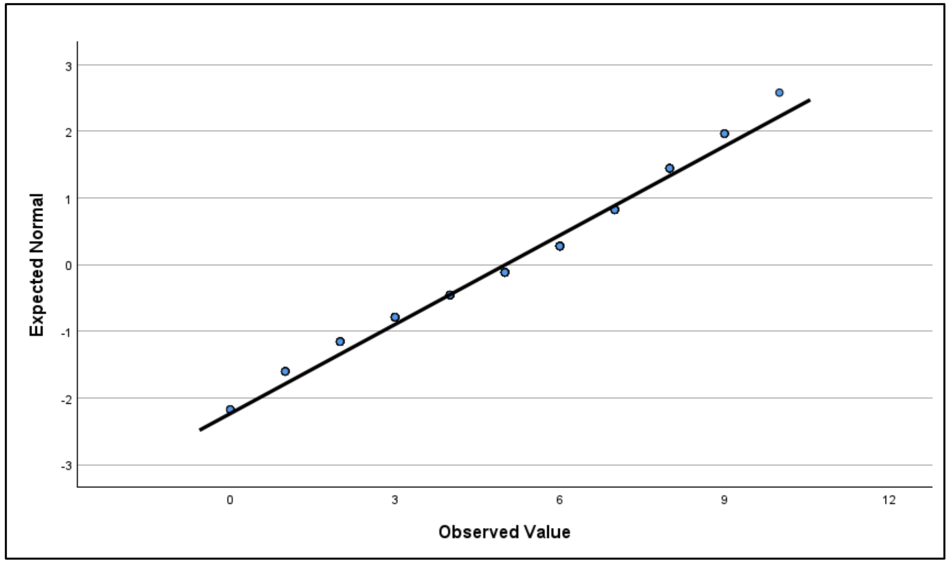

2. Multiple regression model, Step 1: Analysis without organizational predictors

		95% CI			
Step 1 (adj. R^2=17.1%)	B	LL	UL	β	ρ
Personal variables					
Gender	-0.23	-0.88	0.43	-0.05	0.498
Age	0.80	-0.07	1.66	0.13	0.070
Profession	-1.52	-2.17	-0.87	-0.33	<0.001*
Palliative care training (any)	-0.67	-1.33	0.00	-0.14	0.050
Spirituality	0.08	-0.24	0.40	0.04	0.624
Self-care (delimit)	-0.10	-0.55	0.35	-0.04	0.655
Self-care (support opportunities)	0.50	-0.17	0.83	0.24	0.003*
General self-efficacy	0.04	-0.04	0.13	0.07	0.332
Thanatophobia	0.01	-0.04	0.05	0.02	0.796
Burden factors	-0.05	-0.12	0.03	-0.10	0.194

3. Correlation matrix of model variables (personal predictors, organizational predictors, outcome variable (Pearson's correlation (r))

	Outcome	Gender	Age	Profession	Palliative care training	Spirituality	Self-care (delimit)	Self-care (support)	General self-efficacy	Thanatophobia	Burden factors	Type of ward	Interprofessional teamwork[a]
Outcome	1.00												
Gender	0.03	1.00											
Age	**0.14***	-0.08	1.00										
Profession	**-0.34****	**-0.24****	-0.04	1.00									
Palliative care training (any)	-0.09	-0.08	**0.27****	0.13	1.00								
Spirituality	0.04	-0.11	**0.30****	0.02	**0.22****	1.00							
Self-care (delimit)	**0.14***	0.04	0.06	-0.02	**0.26****	-0.06	1.00						
Self-care (support opportunities)	**0.20****	-0.09	0.05	0.10	**0.23****	0.01	**0.53****	1.00					
General self-efficacy	0.14	**0.19****	0.04	-0.12	**0.14***	0.06	**0.31****	**0.20***	1.00				
Thanatophobia	-0.11	0.12	**-0.17***	0.08	**-0.25****	0.02	**-0.46****	**-0.30****	**-0.17***	1.00			
Burden factors	**-0.22****	-0.07	-0.12	**0.25****	-0.08	0.14	**-0.41****	**-0.23****	**-0.26****	**0.34****	1.00		
Type of ward	**0.22****	-0.12	0.01	**-0.19****	-0.07	-0.08	-0.06	-0.03	0.03	0.13	**-0.20****	1.00	
Interprofessional teamwork[a]	**0.45****	-0.08	-0.07	-0.06	-0.04	-0.05	**0.20****	**0.37****	**0.18***	-0.04	**-0.22****	**0.20****	1.00

p<0.05 = *, p<0.001 = **
[a] n=193

4.Test for interaction effects

a. Test for interaction between profession (nurse vs. rest) and type of ward (ICUs vs general wards) regarding the outcome variable (general linear model)

n=201	**F**	**p**
Type of ward (ICU vs. GW)	6.11	0.014
Profession (nurse vs rest)	19.58	<0.001
Type of ward * Profession	0.11	0.741

b. Mean comparison for type of ward (ICUs vs general wards) and profession (nurse vs rest) regarding the outcome

		**Type of ward**	
		ICU	GW
**Profession**	Rest	5.6	6.2
	Nurse	4.1	5.0

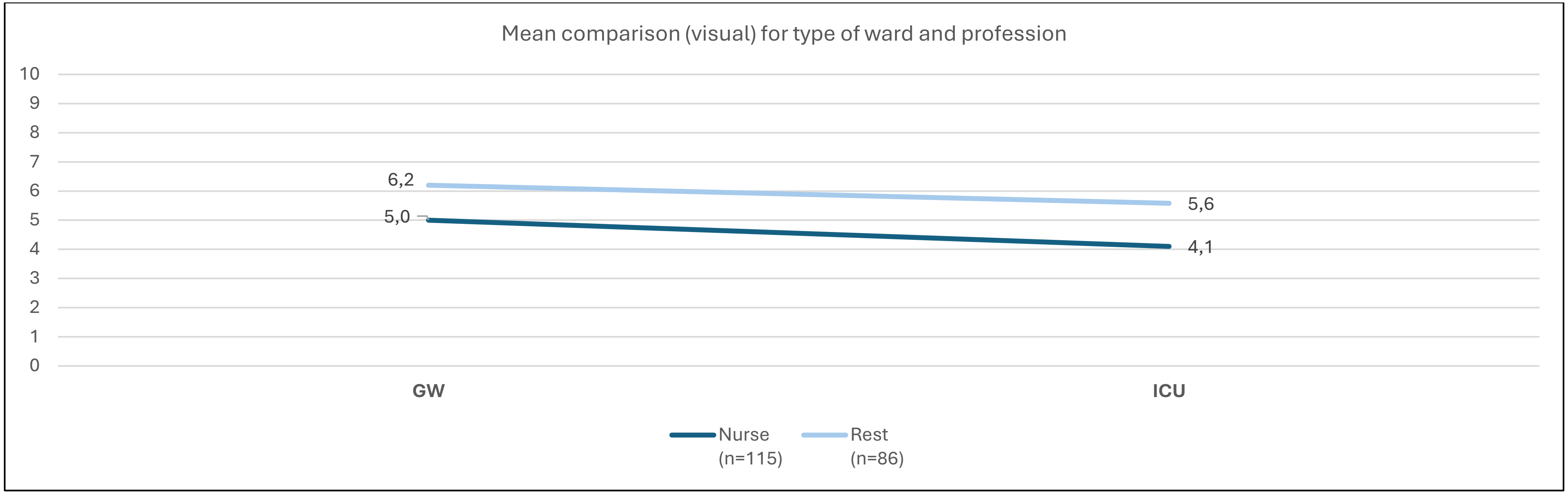

5. Statistic checks (normal distribution of residuals, scatter plot to check homoscedasticity) for the hierarchical multiple regression model (n=193):

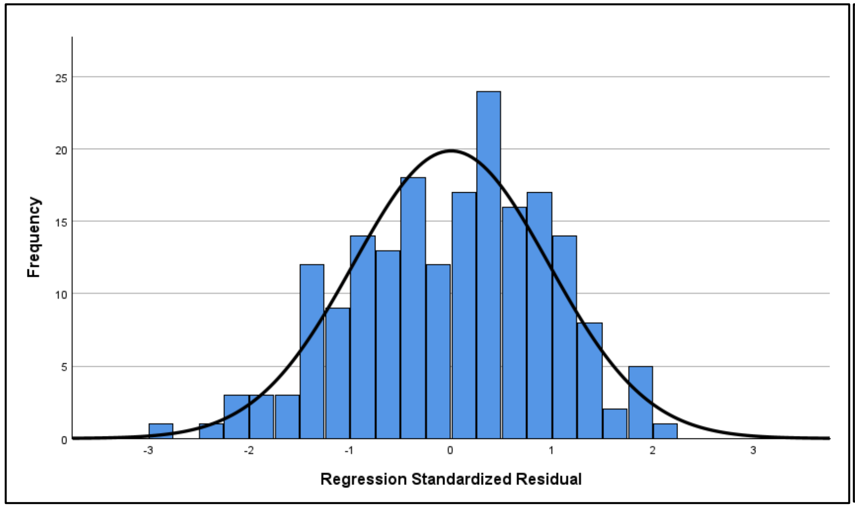

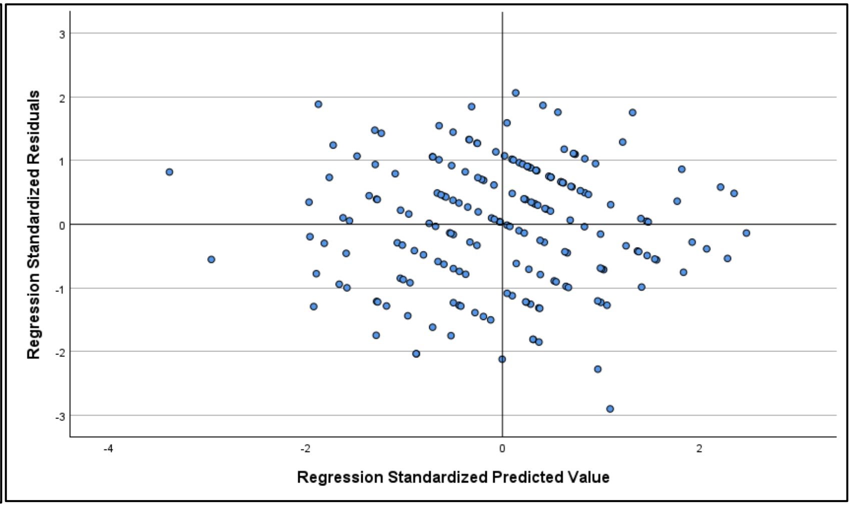

Supplement: S3 Appendix — (PDF) [file pone.0334650.s003.pdf]
