# Supplementary material for: How do healthcare professionals on non-palliative care wards perceive quality of care in the dying phase? Personal and organizational predictors identified in a cross-sectional study
Source: PLoS One. 2025 Oct 31;20(10):e0334650. doi: 10.1371/journal.pone.0334650 (PMC12578199; doi:10.1371/journal.pone.0334650)
Supplement: Attachment — Submitted filename: Peer Review PONE - D24 - 31789.pdf [file pone.0334650.s005.pdf]

Peer Review Assessment

Introduction

1. Recommend not starting the 1st line of the paragraph with “How”.
2. Paragraphs 81 - 88. Would there be a possibility to add more of what the studies had shown to give the reader a more clear picture of the known literature on this topic by including percentages/figures.
3. Reference for line 92 needed
4. Line 95. Would not start with “ While Spiritual care..”. Would benefit from a more structured beginning to the point
5. Paragraph on personal factors - Benefits from a more structured breakdown of personal factors, the reader is moving to different personal factors without connection. Eg Spiritual care is the 1st pont then Gender then age etc.
6. Paragraph Work related organizational factors will need revision for more organized thought explanation and structured listing/explanation of points
7. Line 124 - 126. Could the author expound more on the research done as well as details on the interprofessional teamwork and the benefits given it was found as a strong predictive factor in their own study. Possibly expound what factors/nuances exemplifies good interprofessional work in other studies

Data

1. It would be notable to add this study was done during the covid epidemic earlier in the study rather than just in the limitations
2. No mention of a Power analysis. How was the number of participants needed/sample size calculated and was it powered to determine a difference between ward types? Especially with a 35% response rate
3. Was there AIC/BIC analysis to determine if the Hierarchical Regression model was the best fit for the study. Can a comment on this be mentioned in the analysis for clarity if this was done?
4. What outlier checks were done for extreme values e.g Cook's distance
5. The results for age ($p = 0.02$) indicate statistical significance, but the effect size (Beta = 0.15) appears small. Additionally, the confidence interval is relatively wide, suggesting some uncertainty in the estimate, particularly given the small >50 group (16.9% of the sample). Is dichotomizing the age in an unbalanced fashion giving the most accurate result regarding the impact of age in this study?
6. Regarding the descriptive results section. It may be improved via graphical data rather outlayed in a paragraph to show the Medians and S.D
7. In the descriptive results section, Line 254 - 255. Words such as Moderate and High were used however the reader would not be able to verify that given there was no grading scale explained

8. The p Value of 0.06 between wards needs to be commented on as it is almost significant, was the sample size enough to determine a difference between these groups? More weight should be given to this aspect in the discussion.
   This is important as if there is a difference in wards, it can help where to place resource allocation.
9. The study might benefit from testing for interaction effects between nurse status/profession and ward type (ICU vs general ward). Given the potential for differing experiences or perceptions across these groups, incorporating an interaction term could provide deeper insights into how nurse status influences in different hospital settings

Limitations

1. Was there any comment on the 35% response rate and how this may have affected your initial analysis for your sample. Could there be a response bias
2. Under representation of non nurses as well as more than double the amount of ICU nurses than GW
3. It would also be notable to recommend to revisit the study with new information outside of the covid pandemic given this study may not have generalizable abilities during a non pandemic scenario
