# Supplementary material for: How do healthcare professionals on non-palliative care wards perceive quality of care in the dying phase? Personal and organizational predictors identified in a cross-sectional study
Source: PLoS One. 2025 Oct 31;20(10):e0334650. doi: 10.1371/journal.pone.0334650 (PMC12578199; doi:10.1371/journal.pone.0334650)
Supplement: Attachment — Submitted filename: Oubaid_et_al_PONE_Response_to_reviewers.pdf [file pone.0334650.s008.pdf]

Manuscript ID: PONE-D-24-31789
Manuscript's title: How do healthcare professionals on non-palliative care wards perceive quality of care in the dying phase? Personal and organizational predictors identified in a cross-sectional study
Corresponding Author: Nikolas Oubaid

Response to Reviewers

Dear Editor, dear reviewers,

Thank you for giving my co-authors and me the opportunity to revise our manuscript titled "How do healthcare professionals on non-palliative care wards perceive quality of care in the dying phase? Personal and organizational predictors identified in a cross-sectional study". My co-authors and I appreciate the time and effort that you have dedicated to providing and communicating valuable feedback on our manuscript and suggesting ideas for improvement on a detailed level.

We incorporated changes to reflect most of the suggestions provided by the reviewers and we highlighted the changes within the manuscript (red text for additions, ~~crossed-out gray~~ text for deletions).

We responded to every aspect of the reviewer's feedback point-by-point:

Reviewer #1:

Overall response: Thank you for your detailed feedback, especially on our main outcome and the rationale of our work, helping us to improve the overall quality and readability of our manuscript. We substantially revised the "introduction" and "discussion" chapters regarding your and Reviewer #2's comments. We also added a comprehensive "Analysis Appendix". Thank you for pointing out some typographical errors – we revised the entire manuscript for readability.

Point-by-point response:

Abstract

"This makes their perspective important to improve the quality of care in the dying phase." – perspective on what? Please re-write this sentence.

> Thank you for this hint, we rephrased that sentence and clarified, that we are investigating HCPs' perceptions of the quality of care in the dying phase on their ward (page 2, line no. 55-56).

Methods – What were the predictors?

We added the predictor variables to the abstract (page 2, line no. 63-66). Thank you for this hint!

Results – "The regression model was significant (p<0.001) and explained 30.7% of the total variance." – This sentence is not necessary in the abstract. We don't really know what was in the model.

Thank you for this hint! As we added the predictors to the abstract like you suggested, therefore we let this sentence remain in the abstract.

Discussion and in general: There is an underlying assumption that the perception of healthcare professionals = reality, which is not the case. Why should we explore what predicts healthcare professional's perceptions of quality of care in the dying phase? Why is it worthwhile to have interventions to improve the perceived quality of care in the dying phase, rather than interventions that can improve the quality of care measured with patient-centred outcome measures and establish quality metrics?

Thank you for sharing this very reasonable concern! We agree and there are studies that imply that the perceived quality of care of HCPs does not always equal the quality of other indicators (e.g. quality reported by patients or informal caregivers). However, the perception of HCPs is a significant parameter to improve quality of care, especially in the dying phase (many patients in the dying phase are non-communicative and patient reported outcomes are difficult to measure and collect). HCPs also often experience moral distress when caring for dying patients or experience higher levels of distress in general, when they perceive the quality of a patients' death as low. HCPs' perception is therefore important to create a healthy work environment, keep HCPs engaged and consequently to improve the quality of care in the dying phase.

However, as we see your valuable point we revised the entire "introduction" chapter and added a stringent paragraph regarding the importance of HCPs' perception of the quality of care – especially in the dying phase (page 5-6, line no. 146-178).

<u>Introduction</u>

Page 3, Line 88 – "their perspectives and experiences are highly important to improve quality of care in the dying phase." – But this paper does not explore people's perspectives on how to improve the quality of care for people at the end of life, nor their experiences.

Thank you for pointing this out! We agree that our original reasoning is not specific enough. We rephrased that sentence (and the rest of the "introduction" chapter) to focus on why HCPs' perception of the quality of care in the dying

phase is an important parameter for the healthcare system and to improve quality of care (pages 5-6, line no. 146-178). We also clarified that our outcome is not perspective nor experience, but perception of quality of care in the dying phase.

Page 3, Line 92 – Empirical

Thank you for this hint – we corrected the word (page 4, line 109). We also spell checked the rest of the document.

Please use the introduction section of the manuscript to provide background literature but also to give a rationale for why this study is needed. Listing every single association with the quality of care or attitudes towards end-of-life care (which are not your outcome) is not helpful to the reader. For instance, on page 4, line 104 – "However, the perception between different professions of patient satisfaction and of the quality of care provided can differ [21, 22]." – what does 'different professions of patient satisfaction' mean?

Thank you for this comment - in context of your overarching comment regarding the outcome and the question of whether the perception of quality of care is relevant and in combination with comments from Reviewer #2, we have generally revised the "introduction" chapter (also see your final comment "clinical implications") and enhanced the part about our rationale (page 5-6, line no. 146-178). However, we have retained some empirical studies and reviews of the literature on the predictors of quality of care, as we assessed if these discussed predictors of quality of care (in the dying phase), mostly reported by patients and informal caregivers, are also predictors of HCPs' perception. As we see your point, we revised the original paragraph about the predictor variables as well (pages 4-5, line no. 109-138).
Regarding the "different professions"-comment: We revised that sentence (page 4, line no. 112-113). Thank you for pointing this out!

Page 4, Lines 109- 110: "Palliative care-specific training showed a learning effect and an increase in perceived competence among HCPs, but it is not known whether this translates into better, sustained quality of care [26, 27]." – this paper is not measuring the quality of care.

Thank you for this hint! As we revised the "Introduction" chapter, we also revised this paragraph/sentence accordingly (page 5, line no. 136-138).

Page 5 – Line 133 – GW unnecessary acronym.

We deleted GW as an acronym to increase readability of the manuscript. However, we left it in figures and tables to save space and increase an easy overview of the data.

Page 5 – Lines 133-134: "However, their perception is important, as they are also the providers of care in the dying phase." – Why? Is there evidence of concordance between patient's perception of quality of care and HCPs' perceptions? Are their perceptions correlated with actual care quality that is delivered?

Thank you for this important hint and the question! We revised the entire "Introduction" chapter and pointed out, why HCPs' perception of quality of care in the dying phase is an important parameter and how the perception of quality of care is embedded in clinical practice (page 5-6, line no. 146-178).
We also added literature to support the statement that HCPs' perception of quality of care in the dying phase is a common method to measure quality of care in the dying phase (e.g. a systematic review by Kupeli et al. (2019) mentions the challenges of measuring quality of care in the dying phase and lists tools that are also based on HCPs' perceptions).

Page 5 – Page 135-137: What does "overall quality of care" mean? There is a massive amount of work dedicated to measuring and improving the quality of care towards the end of life. Please support these statements (which are not clear) with references if they are true.

Thank you for this reasonable concern! We used this self-constructed item (overall quality of care) intentionally, because in palliative care, certain single aspects of care (psychological care, intensity of pain or other somatic symptoms, …) are often being used as outcomes (also in the literature we cited) and are part of long and complex item sets. We used this single item as a proxy-variable. We hoped that using a short and single item compared to a longer item set would also increase the motivation of HCPs to finish the survey (out of the 35% response rate, we managed to receive a 79% completion rate).

We also added a statement to the "data analysis" chapter on why we intentionally used the self-constructed item (pages 9-10, line no. 243-246).

As this study needs to be revised on a larger, independent sample we also suggested in the "future research" chapter that this item should be used along established item sets for further validation (page 21, line no. 452-460).

<u>Methods</u>

Page 7 – Lines 174-175: If the authors do not recommend summing up the scores, why did you go ahead and do it anyway? Surely, this means adding the items up does not lead to a meaningful sum.

Thank you for this methodically important question! We rephrased that sentence (page 8, line no. 209-210). In our opinion, the single items of this instrument provide a good representation of the subjectively perceived burden factors that

can occur when caring for dying patients and supporting their informal caregivers as the items represent a checklist of different aspects of burden factors. Additionally, its' Cronbach's Alpha value is above 0.75 in our data as well. Furthermore, these items have already been used on palliative care wards in a larger national study.
However, the point you are making is very important, and this measurement instrument would need to be validated further. We have therefore added a corresponding sentence to the "discussion of non-significant predictors" (page 18, line no. 395-397) and the "strengths and limitations" (page 20, line no. 426-427) chapters.

<u>Data Analysis</u>

Please state which variables were dichotomised and the reasons. Thank you for providing a detailed explanation of the statistics checks. Your final sample seems to be skewed towards people who work in ICUs. There are more general wards in hospitals than ICUs.

Thank you for this suggestion and the hint! We clarified which variables were dichotomized (page 12, line no. 274). We also gave specific reasons (page 12, line no. 276-278).
We expanded our limitations regarding the imbalance of ICU and general ward HCPs in our sample (page 19, line no. 422-424). We also added to the "future research" chapter that this study should not only be repeated outside of pandemic context, but also with a more balanced sample (pages 20-21, line no. 454-457).
We also included an "Analysis Appendix" for further statistical background information.

<u>Results</u>

Do you have any information on the non-respondents and the denominator for the sample?

Thank you for this methodological important question! We have provided available and relevant information regarding non-responders and the samples' denominator in the Appendix (STROBE-Guideline and CHERRIES-Guideline).

Why did you dichotomise the profession?

Thank you for this appropriate question. We dichotomized profession because studies suggest that nurses tend to perceive the quality of care different than other professions. Nurses are also more than 50% of our sample, so we wanted

to proceed with a balanced comparison. We added a corresponding sentence (pages 12, line no. 276-278).

Page 11, Line 249: assess not address

Thank you for this hint – we corrected the word (page 13, line no. 289).

Page 13, fully adjusted model: It may be useful to provide the findings for the minimally adjusted model and univariate correlations as well as the fully adjusted model.

Thank you for this great suggestion! We added a comprehensive "**Analysis Appendix**" (first reference in the manuscript: page 13, line no. 296-297) where we displayed bivariate correlations of the predictor variables and the outcome variable, as well as the minimal model (step one, model without organizational predictors). Readers can therefore now better understand correlational relationships between the predictor variables as well as the two regression models.
We also added the results of the AIC analysis (page 14, line no. 310-311) to give the reader a better understanding of the model selection process.

In table 6 - just checking that the labels weak and moderate associations are not based on the p value? Please make this clearer.

Thank you for the good idea to clarify that by "weak", "moderate" and "strong" we refer to the standardized beta-values. We made this clear by adding the beta-values (pages 15-16, table 6).

<u>Discussion</u>

The discussion section should highlight the main findings from the study, not describe the modelling steps. Please revise the first paragraph.

Thank you for this correct hint! We revised the entire beginning of our "discussion" chapter (page 16, line no. 342-348).

Page 15, line 303 – Please provide references and actually discuss what your findings may mean.

Thank you for this comment, we added a paragraph regarding possible implications of our results (page 17, line no. 355-360).

Page 15, lines 316-318: "With regard to the perceived quality of care in the dying phase, it is possible that younger HCPs compare the care provided on their ward with a more idealistic care they know from their training and education." – Is this necessarily a bad thing? The explanation for this finding seems like a bit of a stretch.

Thank you for noticing this! We revised the wording in a more neutral way (page 17, line no. 371-372). In the context of Reviews #2s' comment regarding this predictor, we also added a sentence regarding the impact of this predictor (page 18, line no. 375-377).

Discussion of non-significant predictors – Another explanation can be that the previous studies did not assess the association between perceived quality of care at the end of life from the HCPs' point of view.

Thank you for this valuable hint! We added a corresponding sentence (page 19, line no. 402-405) and addressed this aspect.

Clinical implications

I am still not convinced what the implication of improving the perception of the quality of care, rather than improving the quality of care.

We rephrased the manuscript to emphasise why it is important to improve HCPs' perception of quality of care in the dying phase (see your previous comments). We hope that our revision of the manuscript resolved your concern.

Reviewer #2:

Overall response: Thank you for your feedback, especially on the readability of our manuscript, as well as on methods and data analysis. Thank you for suggesting the reporting of additional tests to improve the methodological quality of our manuscript. Regarding Reviewer #1 and your comments we also added a comprehensive "**Analysis Appendix**", providing additional information.

Point-by-point response:

Introduction

1. *Recommend not starting the 1st line of the paragraph with "How".*
    a. Thank you for this suggestion, we rephrased the beginning of our manuscript (page 3, line no. 87-88).
2. *Paragraphs 81 - 88. Would there be a possibility to add more of what the studies had shown to give the reader a more clear picture of the known literature on this topic by including percentages/figures.*
    a. This is a very reasonable request! In the context of your other comments regarding the introduction and Reviewer #1's comments, we decided to revise the entire "introduction" chapter. We emphasised why HCPs' perception of quality of care is an important clinical outcome and that we wanted to check whether the discussed predictors of quality of (end-of-

life) care are also predictors of HCPs' perceived quality of care in the dying phase in one model.
   b. As we see your valuable point, we added more details (p-values, better description of outcome variables, ...) whenever possible in the "introduction" chapter (e.g. page 4, line no. 114; page 5, line no. 131).
3. *Reference for line 92 needed*
   a. Thank you for this hint – we rephrased that sentence.
4. *Line 95. Would not start with " While Spiritual care..". Would benefit from a more structured beginning to the point*
   a. Thank you for this hint to increase the readability of that sentence. We rephrased the paragraph (pages 4-5, line no. 109-138) and deleted that sentence.
5. *Paragraph on personal factors - Benefits from a more structured breakdown of personal factors, the reader is moving to different personal factors without connection. E.g. Spiritual care is the 1st point then Gender then age etc.*
   a. Thank you for this great suggestion regarding readability of our manuscript! We restructured the introduction (1. predictors of quality of (end-of-life) care (pages 4-5, line no. 109-138) and 2. The need to assess HCPs perceptions (pages 5-6, line no. 146-178)).
   b. We restructured the previously researched predictors into predictors with a relatively high level of evidence (pages 4-5, line no. 110-129) and predictors with heterogenous results in previous literature (page 5, line no. 130-138).
6. *Paragraph Work related organizational factors will need revision for more organized thought explanation and structured listing/explanation of points*
   a. Thank you for this comment! We rephrased the paragraph regarding the clinical setting (page 4-5, line no. 122-129).
7. *Line 124 - 126. Could the author expound more on the research done as well as details on the interprofessional teamwork and the benefits given it was found as a strong predictive factor in their own study. Possibly expound what factors/nuances exemplifies good interprofessional work in other studies*
   a. Thank you for this hint. We elaborated on the construct of interprofessional patient-centred teamwork in the "introduction" chapter (page 4, line no. 117-121) and added more emphasis on it in the "discussion" chapter (page 17, line no. 355-360).

Data

1. *It would be notable to add this study was done during the covid epidemic earlier in the study rather than just in the limitations*

   a. Thank you for this important hint! In this regard we added a corresponding sentence (page 9, line no. 230) so the reader knows about this circumstance before seeing the “results” chapter.
2. *No mention of a Power analysis. How was the number of participants needed/sample size calculated and was it powered to determine a difference between ward types? Especially with a 35% response rate*
   a. With the sample size obtained, we assumed that we could detect clinically relevant differences between the types of wards (ICUs n=126; general wards n=75). Based on your valuable comment, we conducted a post-hoc power analysis: The post-hoc power analysis showed that with the given sample size and a power of ≥80% and a two-sided alpha of 5% we were able to detect a standardized effect (Cohen's d) of d≥0.41 with unpaired t-test statistics. The reference for this post-hoc power analysis can be found in the appendix (STROBE-guideline) and a corresponding sentence that the study should be repeated with a larger sample is included in the “strengths and limitations” chapter (page 21, line no. 452-455).
3. *Was there AIC/BIC analysis to determine if the Hierarchical Regression model was the best fit for the study. Can a comment on this be mentioned in the analysis for clarity if this was done?*
   a. Thank you for this hint! We calculated AIC for model fit analysis to compare the models methodologically and reported R^2 and adj. R^2. We added a corresponding sentence to the “methods” (page 10, line no. 254-255) and “results” chapters (page 14, line no. 310-311).
4. *What outlier checks were done for extreme values e.g Cook's distance*
   a. Thank you for this question! We calculated Cook’s distance and added a sentence to the “data analysis” chapter with literature reference (page 10, line no. 252-253) and to the “results” chapter (page 14, line no. 314-315).
5. *The results for age (p = 0.02) indicate statistical significance, but the effect size (Beta = 0.15) appears small. Additionally, the confidence interval is relatively wide, suggesting some uncertainty in the estimate, particularly given the small >50 group (16.9% of the sample). Is dichotomizing the age in an unbalanced fashion giving the most accurate result regarding the impact of age in this study?*
   a. Thank you for this relevant question! Reviewer #1 asked a similar question regarding the dichotomization. For the variable “age”, among others, the staff councils of the participating University Medical Centers specified that no categories with small cell sizes should be evaluable (data privacy). For this reason, we intentionally categorized age in the survey construction and were therefore limited in the evaluation in this respect. We decided against a distribution-based dichotomization (e.g. by

median), as we could not do this due to the aggregated data collection of age. Studies indicate that moral distress is higher among younger HCPs. We therefore intentionally decided to test “older” versus “younger” HCPs, being aware that this might compromise the balance of the dichotomized groups.

b. However, as we see your valuable point, we added a corresponding sentence to the discussion of the predictor age (page 18, line no. 375-377).

6. *Regarding the descriptive results section. It may be improved via graphical data rather outlayed in a paragraph to show the Medians and S.D*
   a. Thank you for this great suggestion! As our model contains numerous predictor variables, we believe that there is not enough space to display every single item/scale visually in the main manuscript. However, as we see your very valuable point and agree, we included visual data presentation (frequency distribution, boxplots, Q-Q-diagrams) in a newly developed “Analysis Appendix” regarding the used scales (General self-efficacy, Thanatophobia, Interprofessional patient-centred teamwork, Burden related to care in the dying phase) and the outcome variable. We gave a corresponding hint in the manuscript (first mentioned on page 12, line no. 285).
7. *In the descriptive results section, Line 254 - 255. Words such as Moderate and High were used however the reader would not be able to verify that given there was no grading scale explained*
   a. Thank you for this hint! The scales do not have a categorization (e.g. “small”, “moderate” or “high”). It was our subjective interpretation of the empirical results. We deleted the mentioned wording.
8. *The p Value of 0.06 between wards needs to be commented on as it is almost significant, was the sample size enough to determine a difference between these groups? More weight should be given to this aspect in the discussion. This is important as if there is a difference in wards, it can help where to place resource allocation.*
   a. Thank you for this relevant aspect. This is something my co-authors and I also discussed when we first saw the results. We added a corresponding sentence to the "discussion of non-significant predictors" chapter (pages 18-19, line no. 397-400). We also gave a hint in the “future research” chapter (page 21, line no. 452-459) that a revised study should include an independent, larger sample and be more balanced between ICUs and general wards.
   b. We also tested for an interaction effect between type of ward and profession (as you suggested in your following comment) and included the results in the “Analysis Appendix”.

   c. We also calculated a post-hoc power analysis as mentioned in your previous comment.

9. *The study might benefit from testing for interaction effects between nurse status/profession and ward type (ICU vs general ward). Given the potential for differing experiences or perceptions across these groups, incorporating an interaction term could provide deeper insights into how nurse status influences in different hospital settings*
   a. Thank you for this very interesting thought! We tested a possible interaction effect between profession (nurse vs rest) and type of ward (ICUs vs general wards) in a general linear model. There were no interaction effects (p=0.741 and visual mean comparison) in our data. However, since this is a valuable concern and a good suggestion, we added a sentence in the results chapter (page 15, line no. 325-326) and included the mentioned analysis in the new "analysis appendix".

Limitations

1. *Was there any comment on the 35% response rate and how this may have affected your initial analysis for your sample. Could there be a response bias*
   a. Thank you for this hint! We gave some background information regarding the (detailed) response rate in the Appendix (CHERRIES- and STROBE-Guideline). We also added a sentence in the "discussion" chapter regarding a possible response bias (page 19, line no. 406-410).
2. *Under representation of non nurses as well as more than double the amount of ICU nurses than GW*
   a. Thank you for this valuable hint! We added this point to the "strengths and limitations" (page 19, line no. 420-422) and the "future research" chapters (page 21, line no. 452-459).
3. *It would also be notable to recommend to revisit the study with new information outside of the covid pandemic given this study may not have generalizable abilities during a non pandemic scenario*
   a. Thank you for this valuable hint. We now point out earlier in the manuscript, that data was collected during the COVID-Pandemic (page 9, line no. 230) as you suggested. We also added a corresponding sentence to the "future research" chapter (page 21, line no. 452-459).
