# Supplementary material for: How do healthcare professionals on non-palliative care wards perceive quality of care in the dying phase? Personal and organizational predictors identified in a cross-sectional study
Source: PLoS One. 2025 Oct 31;20(10):e0334650. doi: 10.1371/journal.pone.0334650 (PMC12578199; doi:10.1371/journal.pone.0334650)
Supplement: Attachment — Submitted filename: Revision - PONE - D - 24 - 31789.pdf [file pone.0334650.s007.pdf]

Introduction

1. Written with a large amount of expansion on the background evidence. Writing style remains cumbersome with a vast amount of information which can be concisely written
2. Recommend Highlighting the points which would mirror what is being studied in the paper which will allow smooth flow and exposing which factors the reader should be paying attention to while reading
3. Recommend defining thanatophobia as well

Statistical Analysis

1. Please comment on the power analysis done for the study

Results

1. Line 240. Recommend rephrasing “Worked on ICUs”.

Limitations

1. Low Response rate should be mentioned as a limitation as well

Overall Comment

- Improved from prior original version with much more detailed and rigorous statistical analysis
- Clear explanations of the limitations noted and context of the study which is helpful
- Recommend condensing the introduction as it appears almost in length as the discussion.

Recommendation - Minor Revision once the paper is balanced better
