# Supplementary material for: How do healthcare professionals on non-palliative care wards perceive quality of care in the dying phase? Personal and organizational predictors identified in a cross-sectional study
Source: PLoS One. 2025 Oct 31;20(10):e0334650. doi: 10.1371/journal.pone.0334650 (PMC12578199; doi:10.1371/journal.pone.0334650)
Supplement: Attachment — Submitted filename: Peer review PONE-D-24.docx [file pone.0334650.s004.docx]

Thank you for the opportunity to review this paper. It is a well-written piece of work. However, the rationale for the choice of outcome of interest of this study is not clear.

Abstract

“This makes their perspective important to improve the quality of care in the dying phase.” – perspective on what? Please re-write this sentence.

Methods – What were the predictors?

Results – “The regression model was significant (p<0.001) and explained 30.7% of the total variance.” – This sentence is not necessary in the abstract. We don’t really know what was in the model.

Discussion and in general: There is an underlying assumption that the perception of healthcare professionals = reality, which is not the case. Why should we explore what predicts healthcare professional’s perceptions of quality of care in the dying phase? Why is it worthwhile to have interventions to improve the perceived quality of care in the dying phase, rather than interventions that can improve the quality of care measured with patient-centred outcome measures and establish quality metrics?

Introduction

Page 3, Line 88 – “their perspectives and experiences are highly important to improve quality of care in the dying phase.” – But this paper does not explore people’s perspectives on how to improve the quality of care for people at the end of life, nor their experiences.

Page 3, Line 92 – Empirical

Please use the introduction section of the manuscript to provide background literature but also to give a rationale for why this study is needed. Listing every single association with the quality of care or attitudes towards end-of-life care (which are not your outcome) is not helpful to the reader. For instance, on page 4, line 104 – “However, the perception between different professions of patient satisfaction and of the quality of care provided can differ [21, 22].” – what does ‘different professions of patient satisfaction’ mean?

Page 4, Lines 109- 110: “Palliative care-specific training showed a learning effect and an increase in perceived competence among HCPs, but it is not known whether this translates into better, sustained quality of care [26, 27].” – this paper is not measuring the quality of care.

Page 5 – Line 133 – GW unnecessary acronym.

Page 5 – Lines 133-134: “However, their perception is important, as they are also the providers of care in the dying phase.” – Why? Is there evidence of concordance between patient’s perception of quality of care and HCPs’ perceptions? Are their perceptions correlated with actual care quality that is delivered?

Page 5 – Page 135-137: What does “overall quality of care” mean? There is a massive amount of work dedicated to measuring and improving the quality of care towards the end of life. Please support these statements (which are not clear) with references if they are true.

Methods

Page 7 – Lines 174-175: If the authors do not recommend summing up the scores, why did you go ahead and do it anyway? Surely, this means adding the items up does not lead to a meaningful sum.

Data Analysis

Please state which variables were dichotomised and the reasons. Thank you for providing a detailed explanation of the statistics checks. Your final sample seems to be skewed towards people who work in ICUs. There are more general wards in hospitals than ICUs.

Results

Do you have any information on the non-respondents and the denominator for the sample?

Why did you dichotomise the profession?

Page 11, Line 249: assess not address

Page 13, fully adjusted model: It may be useful to provide the findings for the minimally adjusted model and univariate correlations as well as the fully adjusted model.

In table 6 - just checking that the labels weak and moderate associations are not based on the p value? Please make this clearer.

Discussion

The discussion section should highlight the main findings from the study, not describe the modelling steps. Please revise the first paragraph.

Page 15, line 303 – Please provide references and actually discuss what your findings may mean.

Page 15, lines 316-318: “With regard to the perceived quality of care in the dying phase, it is possible that younger HCPs compare the care provided on their ward with a more idealistic care they know from their training and education.” – Is this necessarily a bad thing? The explanation for this finding seems like a bit of a stretch.

Discussion of non-significant predictors – Another explanation can be that the previous studies did not assess the association between perceived quality of care at the end of life from the HCPs’ point of view.

Clinical implications

I am still not convinced what the implication of improving the perception of the quality of care, rather than improving the quality of care.
