# Supplementary material for: How do healthcare professionals on non-palliative care wards perceive quality of care in the dying phase? Personal and organizational predictors identified in a cross-sectional study
Source: PLoS One. 2025 Oct 31;20(10):e0334650. doi: 10.1371/journal.pone.0334650 (PMC12578199; doi:10.1371/journal.pone.0334650)
Supplement: Attachment — Submitted filename: Oubaid_et_al_PONE_Response_to_reviewers_Minor_Revisions.docx [file pone.0334650.s009.docx]

Manuscript ID: PONE-D-24-31789
Manuscript’s title: How do healthcare professionals on non-palliative care wards perceive quality of care in the dying phase? Personal and organizational predictors identified in a cross-sectional study
Corresponding Author: Nikolas Oubaid

**Response to Editor and Reviewers**

Dear Editor, dear Reviewers,

Thank you for giving my co-authors and me the opportunity to revise our manuscript titled “How do healthcare professionals on non-palliative care wards perceive quality of care in the dying phase? Personal and organizational predictors identified in a cross-sectional study”. My co-authors and I appreciate the time and effort that you have dedicated to providing and communicating valuable feedback on our revised manuscript and suggesting ideas for improvement on a detailed level.

We incorporated changes to reflect most of the suggestions provided by the reviewers, and we highlighted the changes within the manuscript (red text for additions, ~~crossed-out gray~~ text for deletions).

Overall: We have cited a paper that provides a more detailed analysis of the burden factors (page 6, line no. 163-164).

**Reviewer #2:**

Overall comment: *Overall much improvement is noted in this final revision with significant impact regarding the methodology rigor which represents the backbone of this study. Some small grammar errors at noted regarding "Working on ICU" in multiple sections and also not including the power analysis which needs to be commented on.*

*With small minor revisions left, I am impressed with the work placed to pull this paper to be much better.*

Overall response: Thank you very much for taking the time to review our original submission and our revised version. We will address your specific comments below.

Point-by-point response:

**Introduction**

1. *Written with a large amount of expansion on the background evidence. Writing style remains cumbersome with a vast amount of information which can be concisely written*
2. *Recommend Highlighting the points which would mirror what is being studied in the paper which will allow smooth flow and exposing which factors the reader should be paying attention to while reading*
3. *Recommend condensing the introduction as it appears almost in length as the discussion.*
   a. Thank you for your comments and suggestions! The “Introduction” was most affected by the first round of revisions, which is why it is indeed cumbersome in some places.
      Based on your feedback, we have now shortened and condensed the introduction (pages 3-5. Line no. 85-130 in the clean version), while also still considering the original comments from Reviewer #1 (e.g. the necessity of HCPs’ perception). The introduction has been shortened to two pages in total. We also reduced the amount of literature we cite to increase readability and focus on the most relevant aspects.
4. *Recommend defining thanatophobia as well*
   a. Thank you for this good idea – we defined thanatophobia as fear of death and dying (page 4, line no. 113-114).

**Statistical Analysis**

1. *Please comment on the power analysis done for the study*
   a. Thank you for this valuable aspect! We did not initially perform a power analysis for this specific statistical model (Kremeike et al. 2022, Study Protocol), which was a linear regression model with quality of care as the independent variable and 12 predictor variables. However, we specified that we expected a response rate for the staff survey of 50% (Kremeike et al. 2022) (page 7, line no. 173-174). Since we managed to achieve a response rate of 35% (reasons for non-participation are listed in the STROBE-Guideline), we mentioned that we did not reach our expected sample size in our “data collection”-chapter (page 7, line no. 185-186) and in our “strengths and limitations”-chapter (page 17, line no. 359-360).
   b. We also conducted a post-hoc power analysis for the predictor “type of ward” and the outcome “quality of care”: The analysis showed that with the given sample size (ICUs n=126, GWs n=75) and a power of ≥80% and a two-sided alpha of 5% we were able to detect a standardized effect (Cohen's d) of d≥0.41 with unpaired t-test statistics. In our regression model, the transformed effect size (unstandardized B coefficient / standard error of the estimate) for “type of ward” was d=0.31, which unfortunately fell slightly below d=0.41 and thus the “type of ward” factor is not significant. We mentioned this analysis in the “data collection”-chapter (page 7, line no. 186) and added the calculation to the STROBE-Guideline appendix (S2).

**Results**

1. *Line 240. Recommend rephrasing “Worked on ICUs”.*
   a. Thank you for this stylistic hint. We rephrased that in several chapters (e.g. page 17, line no. 358 and 359; page 4, line no. 108; page 9, line no. 222 and 223).

**Limitations**

- *Low Response rate should be mentioned as a limitation as well*
  a. Thank you for this suggestion – we incorporated a corresponding sentence in “strengths and limitations” (page 17, line 359-360).

**Additional Comment**

- *Improved from prior original version with much more detailed and rigorous statistical analysis*
- *Clear explanations of the limitations noted and context of the study which is helpful*

**Reviewer #3:**

Overall response: Thank you for reading and assessing our revised manuscript!

Point-by-point response:

There were no specific issues raised by Reviewer #3.

**Reviewer #4:**

Overall comment: *The article has, thanks to the corrections already made and suggested by the previous reviewers, gained a great deal in quality in all the chapters. The statistics of the article are excellently set, sophisticated.*

Overall response: Thank you very much for agreeing to be an additional reviewer and for reading and evaluating our revised version. Thank you also for appreciating the work that went into the statistical analysis.

Thank you for your comments on the terminology! Due to the suggestion of Reviewer #2, we shortened and rewrote the introduction. Consequently, we deleted some of the sentences you suggested rephrasing. However, we rephrased the remaining aspects you mentioned.

We will address your specific concerns below.

Point-by-point response:

- *Line 131: the term ”predictor of outcome” should be changed to more appropriate expression (like: predictors of quality of care at the end of life”)*
  - Thank you for this suggestion – we altered the sentence.
- *Line 138: the “clinical outcome” should be changed*
  - We changed “clinical outcome” to HCPs’ perceived quality of care.
- *Line 140: “quality of patients death” should be changed to more appropriate expression*
  - This sentence got deleted in the context of the comments made by Reviewer #2.
- *Line 161-163: the sentence ”Monitoring HCP perception………………….HCP well-being”*
  - This sentence got deleted in the context of the comments made by Reviewer #2.
- *Line 258: Outcome variable should be specified (HCP perceived quality of care in the dying?)*
  - Thank you for the suggestion! We changed “outcome variable” to HCPs’ perceived quality of care*.*
- *Line 359: I advise to use more appropriate expression for “outcome measures”*
  - Thank you for comment. We changed “outcome” to “HCPs’ perceived quality of care”.
- *Line 360: I advise to use more appropriate expression for “outcome”*
  - Thank you for comment. We changed “outcome” to “HCPs’ perceived quality of care”.
- *Line 364: Expression “outcome variables” should be change to “variables”*
  - Thank you for your specific suggestion. We altered the terminology accordingly.
- *Line 404: I advise to use more appropriate expression for “outcome” measures*
  - Thank you for your comment – we changed “outcome measure” to “dependent variable”.
- *Line 405: I advise to use more appropriate expression for caregivers reported “outcome”*
  - Thank you for this hint! We now use “Informal caregivers’ perception”.
- *Line 409: I advise to use more appropriate expression for “outcome”*
  - Thank you for comment. We changed “outcome” to “HCPs’ perceived quality of care”.
